# Predictors of total morbidity burden on days 3, 5 and 8 after cardiac surgery

**DOI:** 10.1186/s13741-017-0060-9

**Published:** 2017-02-14

**Authors:** Julie Sanders, Jackie Cooper, Michael G. Mythen, Hugh E. Montgomery

**Affiliations:** 1 St Bartholomew’s Hospital, Barts Health NHS Trust, London, UK; 2 Institute for Sport, Exercise and Health, University College London, 1st Floor 170 Tottenham Court Rd, London, W1T 7HA UK; 3 Centre for Cardiovascular Genetics, University College London, London, UK; 4 Department of Anaesthesia, University College London Hospitals NHS Trust, London, UK

**Keywords:** Risk factor, Cardiac surgery, Morbidity outcome, C-POMS

## Abstract

**Background:**

Post-operative morbidity affects up to 36% of cardiac surgical patients. However, few countries reliably record morbidity outcome data, despite patients wanting to be informed of all the risks associated with surgery. The Cardiac Post-Operative Morbidity Score (C-POMS) is a new tool for describing and scoring (0–13) total morbidity burden after cardiac surgery, derived by noting the presence/absence of 13 morbidity domains on days 3, 5, 8 and 15. Identifying modifiable C-POMS risk factors may suggest targets for intervention to reduce morbidity and healthcare costs. Thus, we explored the association of C-POMS with previously identified predictors of post-operative morbidity.

**Methods:**

A systematic literature review of pre-operative risk assessment models for post-operative morbidity was conducted to identify variables associated with post-operative morbidity. The association of those variables with C-POMS was explored in patients drawn from the original C-POMS study (*n* = 444).

**Results:**

Seventy risk factors were identified, of which 56 were available in the study and 49 were suitable for analysis. Numbers were too few to analyse associations on D15. Thirty-three (67.3%) and 20 (40.8%) variables were associated with C-POMS on at least 1 or 2 days, respectively. Pre-operative albumin concentration, left ventricular ejection fraction and New York Heart Association functional class were associated with C-POMS on all days. Of the 16 independent risk factors, pre-operative albumin and haemoglobin concentrations and weight are potentially modifiable.

**Conclusions:**

Different risk factors are associated with total morbidity burden on different post-operative days. Pre-operative albumin and haemoglobin concentrations and weight were independently predictive of post-operative total morbidity burden suggesting therapeutic interventions aimed at these might reduce both post-operative morbidity risk and health-care costs in patients undergoing cardiac surgery.

**Electronic supplementary material:**

The online version of this article (doi:10.1186/s13741-017-0060-9) contains supplementary material, which is available to authorized users.

## Background

In surgery, post-operative mortality is the most commonly cited outcome variable and to date has been considered the standard measure of quality of care. However, while cardiac operative mortality has fallen (currently 2.1% in the USA (Society of Thoracic Surgeons) and 1.5% in the UK (Bridgewater et al. 2008)), post-operative morbidity remains common affecting between 4.3% (Fortescue et al. 2001) and 36% (Magovern et al. 1996) of cardiac patients and significantly prolonging length of stay (LOS) (Dupuis et al. 2001). Such morbidity has substantial impact on healthcare resources, with the average in-hospital incremental cost of experiencing any complication at $15,468 per patient (Brown et al. 2008). Thus, strategies to identify and reduce post-operative morbidity might reduce both patient well-being and healthcare costs.

However, few countries reliably record morbidity outcome data (Weiser et al. 2008). Previously, morbidity definitions included death (Fortescue et al. 2001), focused on major morbid events only (Huijskes et al. 2003), or used surrogate markers of morbidity (for example post-operative LOS (Magovern et al. 1996; Dupuis et al. 2001)). Likewise, many national cardiac surgical registers collect only 30-day mortality outcome and in some cases hospital LOS. Contrastingly, although the Society of Thoracic Surgeons national database contains 49 variables related to post-operative events, a composite metric aimed at defining post-operative morbidity includes in-hospital death and only five severe specific morbidities (Shahian et al. 2010).

The Cardiac Post-Operative Morbidity Score (C-POMS) (Sanders et al. 2012) is a simple, validated score (0–13) by which to identify and quantify total morbidity burden (TMB) after adult cardiac surgery (Table 1) on multiple post-operative days. We have previously reported that every unit increase in C-POMS is associated with a 1.7, 2.2 and 4.5-day increase in subsequent LOS on days 3 (D3), 5 (D5) and 8 (D8) (Sanders et al. 2012), which has significant associated health-care costs, organisational and resource implications. Efforts to identify the risk factors associated with this post-operative TMB score may not only assist in identifying modifiable risk factors for which therapeutic interventions can be implemented to reduce the post-operative risk, but also serve to potentially further validate C-POMS as a useful clinical tool in cardiac surgery post-operative morbidity assessment.

**Table 1:** The Cardiac Post-Operative Morbidity Score (C-POMS) (as reported in Sanders et al. 2012)

Morbidity type	C-POMS criteria
Pulmonary	Presence of one or more of the following: ▪ New requirement for oxygen or respiratory support (including nebuliser therapy or request for chest physiotherapy on or after D5) ▪ Pleural effusion requiring drainage
Infectious	Presence of one or more of the following: ▪ Currently on antibiotics ▪ Has had a temperature of >38 °C in the last 24 h ▪ Has a white cell count/CRP level requiring in-hospital review or treatment
Renal	Presence of one or more of the following: ▪ Presence of decreased urine output requiring intervention (including IV furosemide) ▪ Increased serum creatinine (>30% from pre-operative level) ▪ Urinary catheter in situ ▪ New urinary incontinence ▪ Serum potassium abnormalities requiring treatment
Gastrointestinal	Presence of one or more of the following: ▪ Unable to tolerate an enteral diet for any reason including nausea, vomiting and abdominal distension ▪ Nasogastric tube ▪ Diagnosis of a gastrointestinal bleed ▪ Diarrhoea
Cardiovascular	Presence of one or more of the following: ▪ The use of inotropic therapy for any cardiovascular cause ▪ Pacing wires (on or after D5) and/or requiring temporary or new permanent pacing ▪ Diagnostic tests or therapy within the last 24 h for any of the following: (1) new MI or ischaemia, (2) hypotension (requiring fluid therapy, pharmacological therapy or omission of pharmacological therapy, (3) atrial or ventricular arrhythmias, (4) cardiogenic pulmonary oedema, thrombotic event (requiring anticoagulation), (5) hypertension (pharmacological therapy or omission of pharmacological therapy)
Neurological	New neurological deficit (including confusion, delirium, coma, lack of coordination, drowsy/slow to wake, poor swallow, blurred vision, sedated, changing loss of consciousness)
Haematological	Presence of one or more of the following: ▪ Untherapeutic INR requiring pharmacological therapy or omission of pharmacological therapy ▪ Requirement for any of the following within the last 24 h: packed erythrocytes, platelets, fresh-frozen plasma, or cryoprecipitate
Wound	Presence of one or more of the following: ▪ Wound dehiscence requiring surgical exploration or drainage of pus from the operation wound with or without isolation of organisms ▪ Chest drains ▪ Wound pain significant enough to require continuing or escalating analgesic intervention
Pain	Postoperative pain significant enough to require parenteral opioids and/or continuing or additional analgesia
Endocrine	New or additional requirements for blood sugar management
Electrolyte	Electrolyte (including sodium, urea, phosphate) imbalance requiring oral or intravenous intervention (not including potassium as included in renal category)
Review	Remaining in hospital for further review, investigation and/or procedure
Assisted ambulation	A new or escalated post-operative requirement for mobility assistance (including wheelchair, crutches, zimmer frame, walking sticks or assistance)

*CRP* C-reactive protein, *IV* intravenous, *MI* myocardial infarction, *INR* international normalised ratio, *OPA* out-patient appointment, *OT* occupational therapy

Thus, we sought to identify risk factors associated with post-operative TMB, as assessed by the C-POMS tool, with an aim of identifying potentially modifiable risk factors that could be therapeutic targets to reduce post-operative cardiac surgical morbidity.

## Methods

The National Research Ethics Committee London-Bentham (Chair Professor David Katz) gave ethics permission for this study (protocol amendment 7) on 6 September 2011 (reference 04/Q0502/73). All patients included in this study gave written informed consent to participate.

### Participants

Patients were drawn from the development and validation of the C-POMS study, detailed elsewhere (Sanders et al. 2012). In brief, patients undergoing any form of adult cardiac surgery (excluding cardiac surgery for a congenital heart condition or a cardiomyopathy) between January 2005 and November 2007 at the Heart Hospital, University College London Hospitals NHS Trust, UK, and who gave written informed consent were eligible for inclusion. Excluded were those <18 years old, undergoing emergency surgery, who were enrolled in clinical intervention trials or who died within 5 days of surgery.

### C-POMS

The development and validation of the C-POMS tool and score is detailed elsewhere (Sanders et al. 2012). In brief, The McMaster Framework (Kirshner and Guyatt, 1985; Guyatt et al. 1992) for constructing and assessing health indices for discriminative instruments, comprising item selection, item scaling, item reduction and determination of reliability and validity processes, was used. The C-POMS represents TMB as a summary score (0–13), derived by noting the presence or absence of 13 morbidity domains on days 3 (D3), 5 (D5), 8 (D8) and 15 (D15) after cardiac surgery (Table 1).

### Pre- and intra-operative clinical data

A protocol development group, comprising 15 representatives from cardiac nursing, surgery, intensive care and anaesthesia, determined the pre-, intra-, and post-operative variables to be collected prior to commencement of the study. In brief, these included demographic details, past and current medical history, coronary heart disease risk factors, routine biochemistry and haematology measurements, anaesthesia details, operative details and a detailed record of the first 24 h in the intensive care unit. Variables were either obtained from the Society of Cardiothoracic Surgery national database or prospectively from the medical and nursing records by a dedicated, experienced research nurse using a standardised proforma.

### Identification and categorisation of potential risk factors

To identify pre-operative risk prediction models of post-operative morbidity following cardiac surgery, a systematic literature review was conducted using the basic framework for conducting systematic reviews from the Centre for Reviews and Dissemination (Dissemination Centre for Reviews and Dissemination 2001). Three methodological quality filters were utilised. Firstly, the study population was defined as an adult population undergoing any form of cardiac surgery (excluding transplantation and grown-up congenital heart surgery); only methodologies that constructed a pre-operative risk assessment tool were included; valid outcomes were mortality and morbidity. There were no exclusions on the basis of the definition of either outcome. Search terms included cardiac surgery score, cardiac surgery risk score, pre-operative risk; cardiac surgery and risk prediction score; cardiac surgery, coronary artery bypass graft (CABG), surgery morbidity and surgery outcome. In addition to publication databases (the National Centre for Biotechnology Information, Entrez retrieval system and the Web of Science ISI Citation Databases), sources of on-going and recently completed studies (The National Research Register, The Cochrane Library of Systematic Reviews) were also interrogated to identify eligible papers. In total, the abstracts of 1067 papers were scrutinised. Backward and forward citation searches were conducted on all identified eligible papers. Overall, a total of 21 pre-operative risk prediction models were identified.

All pre- and intra-operative variables obtained within the C-POMS study were classified with respect to these models into one of two tiers (results in Additional file 1): Tier 1 variables were those which had been associated with morbidity risk in three or more separate papers (significant evidence), while tier 2 variables were those identified in one or two papers (some evidence).

### Statistical methods

Baseline characteristics are presented as mean ± SD or *n* (%). For the univariate analysis, C-POMS is presented as the median score and compared over categories using the Kruskal-Wallis test. The association of continuous variables with C-POMS was assessed using the Spearman rank correlation. For the multivariate analysis, variables with *p* < 0.25 on univariate analysis were considered for inclusion into the models and stepwise regression with backwards elimination and a threshold of *p* < 0.05 was run. Validation of the models was performed by running 1000 bootstrap samples. Variables selected in at least 60% of the bootstrap samples were included in the final model. For the data on all time points combined, the models used were random intercept models with time fitted as a fixed effect.

## Results

### Baseline participant characteristics

Of 748 potentially eligible patients undergoing cardiac surgery, 520 (69.5%) were screened (due to researcher availability) and 464 (89.2%) consented to participate. Fourteen participants subsequently became ineligible, leaving 450 who completed the study. Six participants declined for their data to be used outside the development of C-POMS.

Table 2 summarises participant characteristics. The majority were White British (379, 85.4%), male (351, 79.1%) with a mean age of 66.6 years (range 19–91 years). Most had triple vessel disease (243, 54.7%), a good (>50%) left ventricular ejection fraction (LVEF) (323, 74.1%) and were of moderate mortality risk (mean EuroSCORE 4.1). The majority had elective surgery (69.6%), using cardiopulmonary bypass (412, 93.4%) and stayed on intensive care unit (ICU) for an average of 2.0 days while remaining in the operating hospital for 9.5 days. The observed in-hospital mortality rate was 1.3%. Overall, 444 (100.0%) were in-patients on D1 and D3, 420 (94.6%) on D5, 178 (40.1%) on D8 and 45 (10.1%) on D15. Subsequent risk factor analysis was only appropriate on D3, D5 and D8 due to low numbers on D15.

**Table 2:** Baseline characteristics (*n* = 444). All values *n *(%) unless otherwise stated

	Frequency/mean ± SD
Demographics	
Age (mean/years)	66.6 ± 10.7
Female gender	93 (20.9)
Ethnicity (White British)	379 (85.4)
Medical history	
Renal (dialysis)	7 (1.6)
History of previous MI	148 (33.3)
Re-operation	18 (4.1)
Symptoms	
NYHA Class	
I	115 (26.0)
II	205 (46.3)
III	101 (22.8)
IV	22 (5.0)
Cardiac risk factors	
Smoking	
Current	49 (11.0)
Ex	245 (55.2)
Never	150 (33.8)
Hypertension	303 (68.2)
Hypercholesteraemia	343 (77.4)
Diabetes	103 (23.2)
Body mass index (kg/m^2^)/mean	28.5 ± 5.6
Examination and investigation	
LVEF	
Good	323 (74.1)
Fair	90 (20.6)
Poor	23 (5.3)
Pre-operative risk assessment	
EuroSCORE	4.1 ± 2.8
Intra-operative details	
Operative priority—elective	309 (69.6)
Operation performed	
CABG	299 (67.3)
AVR	61 (13.7)
MVR	10 (2.3)
CABG + AVR	36 (8.1)
CABG + MVR	0 (0.0)
AVR + MVR	3 (0.7)
CABG + AVR + MVR	2 (0.5)
Other	33 (7.4)
Cardiopulmonary bypass used	412 (93.4)
Outcome	
Length of ICU stay (mean/days)	2.0 ± 3.5
Return to theatre	21 (4.8)
Total length of hospital stay (mean/days)	11.8 ± 11.7

#### Tier 1 and 2 analysis

##### Univariate analysis

Fifty-six variables were identified in tiers 1 and 2. The incidence of seven (12.5%) pre-operative variables (cardiogenic shock, catheter-induced coronary closure, intra-aortic balloon pump, intubation, permanent pacemaker, immunosuppressant medications and inotropes) was too small for analysis, resulting in 49 variables (23 tier 1; 26 tier 2) for analysis. Thirty-three of the 49 (67.3%) variables previously identified to be associated with post-operative morbidity were found to be associated with C-POMS on at least one post-operative day (Table 3). Of those, 10 variables were associated with C-POMS summary score on 1 day only, on either D3 (smoking, body mass index, urgency of operation, use of cardiopulmonary bypass, total drainage within the first 12 h and D1 inotropes) or D5 (ethnicity, neurological history, pre-operative heart rate and dialysis). No variables were solely predictive of D8 C-POMS summary score. Twenty variables (40.8%) were associated with C-POMS summary score on 2 days, while 3 variables (pre-operative albumin and New York Heart Association (NYHA) class and LVEF) were associated with C-POMS summary score on all days.

**Table 3:** Univariate analysis: tier 1 and 2 predictors of C-POMS summary score on D3, D5 and D8

Variable	D3 (*n* = 441)		D5 (*n* = 419)		D8 (*n* = 177)	
	Median C-POMS/Rho	*p*	Median C-POMS/Rho	*p*	Median C-POMS/Rho	*p*
Tier 1 variables						
Demographics						
Age	0.188	0.000	0.110	0.025	0.133	0.078
Age quartiles						
1 (0–59)	2.0	0.002	2.0	0.010	2.0	0.300
2 (60–68)	3.0		2.0		3.0	
3 (69–74)	3.0		2.0		4.0	
4 (≥75)	4.0		2.5		3.0	
Age grp 2						
<65	2.0	0.000	2.0	0.044	2.5	0.083
65–74	3.0		2.0		3.0	
≥75	4.0		2.5		3.0	
Age grp 3						
<70	3.0	0.001	2.0	0.426	3.0	0.225
70–79	3.0		2.0		3.0	
>80	5.0		2.0		3.0	
Gender						
M	3.0	0.024	2.0	0.016	3.0	0.215
F	3.0		3.0		2.5	
Medical history						
Diabetes						
Y	4.0	0.000	2.5	0.015	3.0	0.308
N	3.0		2.0		3.0	
Cerebrovascular disease						
Y	4.0	0.028	3.0	0.023	4.0	0.219
N	3.0		2.0		3.0	
Neurological history						
Y	4.0	0.103	3.0	0.016	4.0	0.553
N	3.0		2.0		3.0	
Congestive heart failure						
Y	4.0	0.009	4.0	0.025	4.0	0.310
N	3.0		2.0		3.0	
COPD/lung disease						
Y	4.0	0.005	3.0	0.012	3.0	0.380
N	3.0		2.0		3.0	
Renal disease						
Y	5.0	0.064	5.0	0.001	5.0	0.025
N	3.0		2.0		3.0	
POSSUM ECG						
Normal	3.0	0.000	2.0	0.000	3.0	0.200
Sinus abnormal	5.0		4.0		–	
AF	4.0		3.0		3.0	
Any other abnormal	5.0		2.5		2.0	
Paced						
Y	5.0	0.005	4.0	0.014	2.5	0.875
N	3.0		2.0		3.0	
Dialysis						
Y	5.5	0.073	7.5	0.006	5.5	0.064
N	3.0		2.0		3.0	
Pre-operative measurements						
Body mass index(kg/m^2^)	0.100	0.043	0.077	0.130	0.099	0.206
LVEF						
Good	3.0	0.013	2.0	0.011	3.0	0.013
Fair	3.0		2.0		4.0	
Poor	5.0		5.0		4.0	
Intra-operative						
Type of surgery						
CABG	3.0	0.000	2.0	0.000	3.0	0.718
AVR	4.0		3.0		3.0	
MVR	5.0		3.0		3.5	
CABG + AVR	4.0		2.0		3.0	
AVR + MVR	3.0		3.0		4.5	
CABG + MVR + AVR	5.0		5.0		6.0	
Other	5.0		3.0		3.0	
Urgency of op						
Elective	3.0	0.002	2.0	0.061	3.0	0.327
Urgent	4.0		2.0		3.0	
Within first 12 h						
Systolic blood pressure (highest)	0.002	0.959	0.026	0.599	−0.089	0.238
Tier 2 variables						
Demographics						
Ethnicity						
Caucasian	3.0	0.052	2.0	0.008	3.0	0.532
Asian	2.0		1.0		3.0	
Black	5.0		3.0		3.0	
Other	2.0		3.0		1.0	
Medical history						
Smoking						
Current	2.0	0.048	2.0	0.395	3.0	0.753
Ex	3.0		2.0		3.0	
Never	3.0		2.0		3.0	
Family Hx CAD						
Y	3.0	0.002	2.0	0.019	3.0	0.686
N	4.0		2.0		3.0	
Atrial arrhythmia						
Y	4.0	0.021	3.0	0.007	3.0	0.781
N	3.0		2.0		3.0	
Pre-operative measurements						
Albumin	−0.210	0.000	−0.144	0.004	−0.183	0.019
Haemoglobin	−0.279	0.000	−0.180	0.000	−0.143	0.058
Heart rate	0.090	0.058	0.179	0.000	0.080	0.294
Weight	0.044	0.369	0.042	0.399	0.101	0.186
No diseased vessels						
0	4.0	0.011	3.0	0.000	3.0	0.203
1	3.0		2.0		2.0	
2	3.0		2.0		2.0	
3	3.0		2.0		3.0	
NYHA class						
1	2.0	0.001	1.0	0.002	3.0	0.001
2	3.0		2.0		3.0	
3	4.0		3.0		3.0	
4	5.0		2.5		5.0	
CCSC class						
0	3.0	0.041	3.0	0.042	3.0	0.317
1	2.0		2.0		2.0	
2	3.0		2.0		3.0	
3	3.0		2.0		3.0	
4	4.0		2.0		4.0	
Cardiomegaly						
Y	5.0	0.000	3.0	0.000	4.0	0.141
N	3.0		3.0		3.0	
Not stated	3.0		3.0		3.0	
Extracardiacarteriopathy						
Y	5.0	0.003	3.0	0.004	4.5	0.147
N	3.0		2.0		3.0	
Current medications						
Diuretic						
Y	4.0	0.003	3.0	0.003	3.0	0.780
N	3.0		2.0		3.0	
Intra-operative						
Cardiopulmonary bypass						
Y	3.0	0.001	2.0	0.072	3.0	0.098
N	2.0		1.0		1.0	
Within 1st 12 h after surgery						
Total drainage	0.139	0.004	0.030	0.542	0.031	0.679
Post-operative (D1)						
Heart rhythm						
Sinus rhythm	3.0	0.000	2.0	0.002	3.0	0.473
Sinus tachycardia	2.5		2.0		2.5	
Sinus bradycardia	4.0		2.0		4.0	
Atrial fibrillation	5.0		3.0		4.0	
Other	3.0		2.0		3.0	
Inotropes						
Y	4.0	0.004	3.0	0.070	3.0	0.923
N	3.0		2.0		3.0	

Overall, 8/23 (34.7%) tier 1 variables and 8/26 (34.7%) tier 2 variables were not associated with C-POMS summary score on any post-operative day (results in Additional file 2).

##### Multivariate analysis

Of the 49 variables, 16 (32.7%) were independent risk factors of C-POMS summary score on one or more post-operative days, while no variables were associated with C-POMS outcome on all three post-operative days (Table 4).

**Table 4:** Tier 1 and 2 (combined) independent predictors of C-POMS summary score

Variable	D3			D5			D8			Combined over all time points	
	Effect	B(se)	*p*	Effect	B(se)	*p*	Effect	B(se)	*p*	B(se)	*p*
Pre-operative haemoglobin	1 SD increase	−0.42 (0.11)	<0.0001		−0.29 (0.12)	0.01					
12-h total drainage	1 SD increase	0.47 (0.10)	<0.001		0.35 (0.10)	0.001				0.31 (0.09)	0.001
Pre-operative weight	1 SD increase	0.34 (0.10)	0.001		0.27 (0.11)	0.02				0.26 (0.09)	0.004
Extra-cardiac arteriopathy	Yes: no	0.73 (0.34)	0.03		0.87 (0.35)	0.01				0.66 (0.31)	0.03
Age	Per year	0.03 (0.009)	0.001							0.024 (0.009)	
Diabetes	Yes: no	0.75 (0.23)	0.002		0.64 (0.27)	0.02				0.92 (0.22)	<0.0001
Left ventricular ejection fraction	Poor: good/fair	0.88 (0.43)	0.04		1.38 (0.45)	0.003					
CABG surgery	Yes: no	−1.17 (0.24)	<0.0001		−1.02 (0.25)	<0.0001				−1.06 (0.21)	<0.0001
MVR surgery	Yes: no	1.40 (0.54)	0.01								
Operative urgency	Urgent: elective	0.48 (0.22)	0.03								
Chronic obstructive pulmonary disease	Yes: no	0.77 (0.30)	0.01								
NYHA class				1 category increase	0.47 (0.24)	0.05					
Renal				Yes: no	2.46 (0.74)	0.001					
Pre-operative cardiomegaly				Yes: no	0.66 (0.32)	0.04				0.66 (0.28)	0.02
Pre-operative albumin							1 SD increase	−0.43 (0.20)	0.04	−0.38 (0.09)	<0.0001
Number of diseased vessels							Increase of 1	1.04 (0.36)	0.005		
Number of saphenous vein grafts							1 SD increase	−0.66 (0.31)	0.04		

There were eight tier 1 variables, all of which (with the exception of renal dysfunction) were associated with TMB on D3. Diabetes, LVEF, CABG surgery and renal dysfunction were also associated with D5 C-POMS summary score. However, only tier 2 variables (pre-operative albumin, number of diseased vessels and number of saphenous vein grafts) were independently predictive of D8 score and these variables were not independent risk factors for any other post-operative day. Pre-operative haemoglobin, 12 h drainage, pre-operative weight and extra cardiacarteriopathy were tier 2 risk factors associated with D3 and D5 morbidity score, while pre-operative cardiomegaly and NYHA class were associated with D5 score only. Considering all time points, 12-h total drainage, pre-operative weight, extra-cardiac arteriopathy, age, diabetes, CABG surgery, pre-operative cardiomegaly and pre-operative albumin level were independently predictive of C-POMS-defined post-operative morbidity outcome.

## Discussion

We found that over two thirds (67.3%) of variables, previously reported as a risk factor for post-operative morbidity, were associated with the new C-POMS (denoting TMB) on at least one post-operative day, with 40% being significant risk factors for two post-operative days and three (6.1%) associated on all three post-operative days. From these results, there are four main findings of note. Firstly, we aimed to identify independent modifiable C-POMS risk factors amenable to therapeutic intervention. These were found to be pre-operative albumin and haemoglobin levels and weight—all tier 2 variables. Previously, pre-operative hypoalbuminaemia has been identified as a risk factor for post-operative delirium (Rudolph et al. 2009), reoperation for bleeding (Engelman et al. 1999), requirement for renal replacement therapy need (Engelman et al. 1999; Sato et al. 2015), increased ICU and hospital LOS (Engelman et al. 1999; Koertzen et al. 2013) and increased mortality (Engelman et al. 1999; Koertzen et al. 2013) cardiac surgery. While hypoalbuminaemia may be directly harmful, it may also mark other pathological states, such as anaemia, since pre-operative hypoalbuminaemia and anaemia are independently associated (Carrascal et al. 2010). Similarly, in cardiac surgery, pre-operative anaemia is associated with in-hospital (De Santo et al. 2009; Hung et al. 2011) or 30-day (Boening et al. 2011) mortality, post-operative blood transfusion rate (De Santo et al. 2009; Hung et al. 2011; Boening et al. 2011), ICU (De Santo et al. 2009; Hung et al. 2011) and in-hospital LOS (De Santo et al. 2009), major adverse cardiovascular events (Boening et al. 2011), and renal complications (De Santo et al. 2009; Boening et al. 2011) than non-anaemic patients. However, evidence relating to whether pre-operative anaemia is associated with infection (Boening et al. 2011) or not (De Santo et al. 2009) is conflicting. There is less evidence relating pre-operative weight to post-operative outcome. Weight loss after bariatric surgery improves hypertension, diabetes and dyslipidaemia (Batsis et al. 2007), although unintended pre-operative weight loss (≥10%) is also associated with prolonged hospital LOS (van Venrooij et al. 2008). Evidence of association is, of course, not the same as proof of causation. However, overall, taken with associated literature, our findings suggest pre-operative albumin, haemoglobin and weight to be candidates for pre-operative interventional studies with the aim of improving post-operative morbidity.

Secondly, we identified 17 *independent* risk factors for TMB on at least one post-operative day. Interestingly, except for renal dysfunction, the other seven tier 1 variables were all associated with D3 morbidity, with only three (diabetes, LVEF and CABG surgery) also being associated on D5 and none with D8 morbidity. Independent risk factors for D8 TMB lay entirely in tier 2. Such findings perhaps suggest that different risk factors are associated with outcome on different post-operative days and that well-accepted risk factors may only be useful for predicting morbidity risk in the first few days of recovery. Patterns of morbidity are well-recognised to differ with time after surgery, and it is likely that their drivers (whether they be intrinsic, environmental, or related to nascent morbidities) will also vary. Such factors are likely to explain the fact that correlations between D3 and D5 with D8 data differed. Furthermore, this study adds to the evidence that pre-operative haemoglobin concentration, pre-operative weight, extra-cardiac arteriopathy, pre-operative cardiomegaly, NYHA class, pre-operative albumin, number of diseased vessels, and number of saphenous vein grafts are independently associated with morbidity outcome.

Thirdly, as expected, the majority of tier 1 variables (those with significant evidence of association with post-operative morbidity) were associated with TMB on at least one post-operative day. While this is not clinically surprising, it does further validate C-POMS as a useful clinical tool in outcome assessment following cardiac surgery. In relation to the eight tier 1 variables that were not associated with TMB on any post-operative day, combining cerebrovascular accident and transient ischaemic attack to a combined cerebrovascular disease variable was associated with a higher C-POMS on D3 and D5, in line with other morbidity risk assessment models (Magovern et al. 1996; Huijskes et al. 2003; Higgins et al. 1992; Tuman et al. 1992)^.^ Furthermore, while hypertension has been independently associated with post-operative morbidity in some studies (for example, Fortescue et al. (2001) and Ivanov et al. (2006)), this has been disputed by others (Higgins et al. 1992; Hattler et al. 1994). However, our results pertaining to systolic blood pressure, previous cardiac surgery and diagnosed peripheral vascular disease are at odds with the literature.

Finally, this study also adds to the more limited data relating to some variables (those in tier 2) and their association with post-operative morbidity after cardiac surgery. Of these, 65.4% were associated with C-POMS TMB score on at least one post-operative day, while pre-operative albumin measurement and NYHA class were risk factors for all three post-operative days. Furthermore, this study also confirmed the findings of previous studies suggesting that unstable angina or recent myocardial infarction (Tuman et al. 1992; Hattler et al. 1994) and pre-existing liver disease (Higgins et al. 1992) are not associated with post-operative morbidity. However, while our study corroborated that of Magovern et al. (Magovern et al. 1996) showing that pre-operative atrial arrhythmia and cardiomegaly are associated with morbidity outcome (Magovern et al. 1996), such results conflict with those found by Hattler and colleagues (Hattler et al. 1994).

There are four main limitations of this study. Firstly, there was no consistent definition of post-operative morbidity used in the pre-operative risk assessment models reported by others. Thus, a wide-range of variables to predict such diversely described outcomes were identified. However, over two-thirds of all variables were found to be associated with TMB, as defined by C-POMS, on at least one post-operative day suggesting that C-POMS is a valid measure of morbidity. Secondly, from the pre-operative risk assessment models, it is difficult to assess what variables were not found to be associated with post-operative morbidity. Most studies did not report variables for which no association with morbidity was identified, due to the often large number (>100) of variables included. We have made our statistically non-significant results available to redress this balance and to aid in the evaluation of this (and other) tools. Thirdly, 14 risk factors identified in the pre-operative risk assessment models were available within this study dataset. Some variables, such as transplantation and ventricular-septal defect, were not available as these surgery types were not included in the study, while the others were non-routinely recorded items. Aside from catastrophic states, the variables not included were all tier 2 variables and in the main were only associated with outcome in one previous study. Finally, analysis to identify risk predictors for C-POMS TMB on D15 could not be conducted due to there being too few participants remaining in the hospital on D15 (*n* = 45). However, this could be the subject of future work.

## Conclusions

Post-operative morbidity is increasingly accepted as an independent quality of care indicator, with approximately 80% of patients wanting to be informed of all the risks associated with surgery (Larobina et al. 2007). Thus, in obtaining operative consent, patients should be told about ‘less serious side effects and complications’ (General Medical Council 2008). C-POMS is a tool that permits such morbidity assessment and TMB scoring at several time points after cardiac surgery, allowing both broad and detailed tracking of morbidities. We have found that pre-operative albumin, haemoglobin and weight are potentially modifiable risk factors for which the investigation of the effect of therapeutic interventions on C-POMS outcome is warranted. Furthermore, we have identified, for the first time, that risk factors differ for different post-operative days. Further work should include the identification of novel risk factors of C-POMS TMB score for each post-operative day, and the identification of risk factors associated with each C-POMS morbidity type to identify the risk factors associated with D15 C-POMS summary score. Additionally, further validation of these results could be sought in a subsequent C-POMS dataset. Such identification of risk factors for C-POMS TMB may aid patient group and individual risk stratification and potentially reduce healthcare costs.

## Additional files

Additional file 1: Variables included in pre-operative prediction models of post-operative morbidity. (DOCX 241 kb)
Additional file 2: Univariate analysis: tier 1 and 2 not predictive of C-POMS summary score on D3, D5 and D8. (DOCX 20 kb)

## Abbreviations

AVR: Aortic valve replacement
CABG: Coronary artery bypass graft
CAD: Coronary artery disease
CCSC: Canadian cardiovascular score
COPD: Chronic obstructive pulmonary disease
C-POMS: Cardiac Post-Operative Morbidity Score
CRP: C-reactive protein
D3 (D5, D8, D15),: Day 3, (day 5, day 8, day 15)
ICU: Intensive care unit
INR: International normalised ratio
IV: Intravenous
LOS: Length of stay
LVEF: Left ventricular ejection fraction
MI: Myocardial infarction
MVR: Mitral valve replacement
NYHA: New York Heart Association
OPA: Out-patient appointment
OT: Occupational therapy
TMB: Total morbidity burden
UK: United Kingdom
USA: United States of America
