# Supplementary material for: Predictors of total morbidity burden on days 3, 5 and 8 after cardiac surgery
Source: Perioper Med (Lond). 2017 Feb 14;6:2. doi: 10.1186/s13741-017-0060-9 (PMC5307860; doi:10.1186/s13741-017-0060-9)
Supplement: Additional file 1: — Variables included in pre-operative prediction models of post-operative morbidity. (DOCX 241 kb) [file 13741_2017_60_MOESM1_ESM.docx]

**Supplementary Table 1: Variables included in pre-operative risk prediction models of post-operative morbidity**

| **Variables** | **Associated with post-operative morbidity** | | | **Not associated with post-operative morbidity** |
| --- | --- | --- | --- | --- |
| **Tier 1** | | | | |
| Age | Pitkanen et al 2000 [[1](#_ENREF_1)], Wouters et al 2002 [[2](#_ENREF_2)], Huijskes et al 2003 [[3](#_ENREF_3)], Parsonnet et al 1989 [[4](#_ENREF_4)], Roques et al 1995 [[5](#_ENREF_5)], Higgins et al 1992 [[6](#_ENREF_6)], Tuman et al 1992 [[7](#_ENREF_7)] , Tu et al 1995 [[8](#_ENREF_8)], Kurki and Kataja 1996 [[9](#_ENREF_9)], Higgins et al 1997 [[10](#_ENREF_10)], Magovern et al 1996 [[11](#_ENREF_11)], Fortescue et al 2001 [[12](#_ENREF_12)], Geraci et al 1993 [[13](#_ENREF_13)], Tremblay et al 1993 [[14](#_ENREF_14)], Eagle et al 1999 [[15](#_ENREF_15)], Wong et al1999 [[16](#_ENREF_16)], Ivanov 2006 [[17](#_ENREF_17)] | | | Hattler et al 1994 [[18](#_ENREF_18)] |
| Body mass index | Kurki and Kataja 1996 [[9](#_ENREF_9)], Magovern et al 1996 [[11](#_ENREF_11)], Staat et al 1999 [[19](#_ENREF_19)], Tremblay et al 1993[[14](#_ENREF_14)], Hattler et al 1994 [[18](#_ENREF_18)] | | | Tuman et al 1992 [[7](#_ENREF_7)]; Kurki and Kataja 1996 [[9](#_ENREF_9)] |
| Cardiogenic shock | Hattler et al 1994 [[18](#_ENREF_18)], Magovern et al 1996 [[11](#_ENREF_11)], Fortescue et al 2001 [[12](#_ENREF_12)] | | |  |
| Cerebrovascular disease/CVA/TIA | Higgins et al 1992 [[6](#_ENREF_6)], Tuman et al 1992 [[7](#_ENREF_7)], Magovern et al 1996 [[11](#_ENREF_11)], Pitkanen et al 2000 [[1](#_ENREF_1)], Fortescue et al 2001 [[12](#_ENREF_12)], Huijskes et al 2003 [[3](#_ENREF_3)] | | | Hattler et al 1994[[18](#_ENREF_18)] |
| Congestive heart failure | Tuman et al 1992 [[7](#_ENREF_7)], Tremblay et al 1993 [[14](#_ENREF_14)], Magovern et al 1996 [[11](#_ENREF_11)], Ivanov et al 2006 [[17](#_ENREF_17)] | | |  |
| Chronic obstructive pulmonary disease | Higgins et al 1992 [[6](#_ENREF_6)], Geraci et al 1993 [[13](#_ENREF_13)], Eagle et al 1999 [[15](#_ENREF_15)], Staat et al 1999 [[19](#_ENREF_19)], Forescue et al 2001 [[12](#_ENREF_12)] | | | Hattler et al 1994 [[18](#_ENREF_18)], Tuman et al 1992 [[7](#_ENREF_7)] |
| Creatinine | Higgins et al 1992 [[6](#_ENREF_6)], Kurki and Kataja 1996 [[9](#_ENREF_9)], Higgins et al 1997 [[10](#_ENREF_10)], Fortescue et al 2001 [[12](#_ENREF_12)], Huijskes et al 2003 [[3](#_ENREF_3)] | | |  |
| Diabetes | Parsonnet et al 1989 [[4](#_ENREF_4)], Kurki and Kataja 1996 [[9](#_ENREF_9)], Pitkanen et al 2000 [[1](#_ENREF_1)], Wouters et al 2002 [[2](#_ENREF_2)], Higgins et al 1992 [[6](#_ENREF_6)], Magovern et al 1996 [[11](#_ENREF_11)], Ivanov et al 2006 [[17](#_ENREF_17)] | | | Tuman et al 1992 [[7](#_ENREF_7)] |
| Electrocardiogram abnormalities | Kurki and Kataja 1996 [[9](#_ENREF_9)], Staat et al 1999 [[19](#_ENREF_19)], Janssen et al 2004 [[20](#_ENREF_20)] | | |  |
| Emergency, urgent, elective surgery | Higgins et al 1992 [[6](#_ENREF_6)], Tuman et al 1992 [[7](#_ENREF_7)], Geraci et al 1993 [[13](#_ENREF_13)], Tremblay et al 1993 [[14](#_ENREF_14)], Kurki and Kataja 1996 [[9](#_ENREF_9)], Magovern et al 1996 [[11](#_ENREF_11)], Eagle et al 1999 [[15](#_ENREF_15)], Wong et al1999 [[16](#_ENREF_16)], Pitkanen et al 2000 [[1](#_ENREF_1)], Fortescue et al 2001 [[12](#_ENREF_12)], Huijskes et al 2003 [[3](#_ENREF_3)], Janssen et al 2004[[20](#_ENREF_20)], Ivanov et al 2006 [[17](#_ENREF_17)] | | | Hattler et al 1994 [[18](#_ENREF_18)] |
| Gender | Parsonnet et al 1989 [[4](#_ENREF_4)], Geraci et al 1993 [[13](#_ENREF_13)], Tuman et al 1995 [[7](#_ENREF_7)], Tu et al 1995 [[8](#_ENREF_8)], Magovern et al 1996 [[11](#_ENREF_11)], Eagle et al 1999 [[15](#_ENREF_15)], Wong et al1999 [[16](#_ENREF_16)], Pitkanen et al 2000 [[1](#_ENREF_1)], Fortescue et al 2001 [[21](#_ENREF_21)], Wouters et al 2002 [[2](#_ENREF_2)], Huijskes et al 2003 [[3](#_ENREF_3)], Ivanov et al 2006 [[17](#_ENREF_17)] | | | Hattler et al 1994 [[18](#_ENREF_18)], Kurki and Kataja 1996 [[9](#_ENREF_9)] |
| Hypertension (history of, systolic blood pressure) | Parsonnet et al 1989 [[4](#_ENREF_4)], Fortescue et al 2001 [[12](#_ENREF_12)] and Wouters et al 2002 [[2](#_ENREF_2)], Ivanov et al 2006 [[17](#_ENREF_17)] | | | Hattler et al [[18](#_ENREF_18)], Higgins et al 1992 [[6](#_ENREF_6)] |
| Lung disease | Kurki and Kataja 1996 [[9](#_ENREF_9)], Wouters et al 2002 [[2](#_ENREF_2)], Janssen et al 2004 [[20](#_ENREF_20)] | | |  |
| Left ventricular dysfunction | Parsonnet et al 1989 [[4](#_ENREF_4)], Higgins et al 1992 [[6](#_ENREF_6)], Tremblay et al 1993 [[14](#_ENREF_14)], Hattler et al 1994 [[18](#_ENREF_18)], Tuman et al 1992 [[7](#_ENREF_7)], Tu et al 1995 [[8](#_ENREF_8)], Roques et al 1995 [[5](#_ENREF_5)], Magovern et al 1996 [[11](#_ENREF_11)], Wong et al1999 [[16](#_ENREF_16)], Eagle et al 1999 [[15](#_ENREF_15)], Pitkanen et al 2000 [[1](#_ENREF_1)], Fortescue et al 2001 [[12](#_ENREF_12)], Wouters et al 2002 [[2](#_ENREF_2)], Huijskes et al 2003 [[3](#_ENREF_3)], Ivanov et al 2006 [[17](#_ENREF_17)] | | |  |
| Myocardial infarction (previous, type, time) | Geraci et al [[13](#_ENREF_13)], Wong et al1999 [[16](#_ENREF_16)], Pitkanen et al 2000 [[1](#_ENREF_1)], Roques et al 1995 [[5](#_ENREF_5)], Hattler et al 1994 [[18](#_ENREF_18)]), Pitkanen et al 2000 [[1](#_ENREF_1)], Huijskes et al 2003 [[3](#_ENREF_3)], Ivanov et al 2006 [[17](#_ENREF_17)] | | | Higgins et al 1992 [[6](#_ENREF_6)] |
| Peripheral vascular disease (history of, previous op or angioplasty) | Magovern et al 1996 [[11](#_ENREF_11)], Eagle et al 1999 [[15](#_ENREF_15)], Higgins et al 1997 [[10](#_ENREF_10)], Ivanov et al 2006 [[17](#_ENREF_17)] | | |  |
| Renal disease/failure (history of/dialysis) | Parsonnet et al 1989 [[4](#_ENREF_4)], Wouters et al 2002 [[2](#_ENREF_2)], Tuman et al 1995 [[7](#_ENREF_7)], Magovern et al 1996 [[11](#_ENREF_11)], Roques et al 1995 [[5](#_ENREF_5)], Pitkanen et al 2000 [[1](#_ENREF_1)], Eagle et al 1999 [[15](#_ENREF_15)], Ivanov et al 2006 [[17](#_ENREF_17)] | | | Hattler et al 1994 [[18](#_ENREF_18)] |
| Re-operation (history of, previous CABG etc) | Parsonnet et al 1989 [[4](#_ENREF_4)], Higgins et al 1992 [[6](#_ENREF_6)], Tuman et al 1992 [[7](#_ENREF_7)], Geraci et al 1993 [[13](#_ENREF_13)], Tremblay et al 1993 [[14](#_ENREF_14)]), Roques et al 1995 [[5](#_ENREF_5)], Tu et al 1995 [[8](#_ENREF_8)], Magovern et al 1996 [[11](#_ENREF_11)], Higgins et al 1997 [[10](#_ENREF_10)], Eagle et al 1999 [[15](#_ENREF_15)], Staat et al 1999 [[19](#_ENREF_19)], Fortescue et al 2001 [[12](#_ENREF_12)], Wouters et al 2002 [[2](#_ENREF_2)], Huijskes et al 2003 [[3](#_ENREF_3)], Janssen et al 2004 [[20](#_ENREF_20)], Ivanov et al 2006[[17](#_ENREF_17)] | | | Hattler et al 1994 [[18](#_ENREF_18)] |
| Type of surgery | Parsonnet et al [[4](#_ENREF_4)], Higgins et al 1992 [[6](#_ENREF_6)], Roques et al 1995 [[5](#_ENREF_5)], Tu et al 1995 [[8](#_ENREF_8)], Pitkanen et al 2000 [[1](#_ENREF_1)], Huijskes et al 2003 [[3](#_ENREF_3)],Ivanov et al 2006 [[17](#_ENREF_17)] | | |  |
| **Tier 2** | | | | |
| Previous myocardial infarction (type and time) | | Tuman et al 1992 [[7](#_ENREF_7)] | |  |
| Albumin | | Magovern et al 1996 [[11](#_ENREF_11)], Higgins et al 1997 [[10](#_ENREF_10)] | |  |
| Anaemia | | Magovern et al 1996 [[11](#_ENREF_11)], Higgins et al 1992 [[6](#_ENREF_6)] | |  |
| Aortic dissection | | Roques et al 1995 [[5](#_ENREF_5)] | |  |
| Atrial arrhythmia | | Magovern et al 1996 [[11](#_ENREF_11)] | | Hattler et al (univariate) [[18](#_ENREF_18)] |
| Body surface area | | Higgins et al 1997 [[10](#_ENREF_10)] | |  |
| Cardiomegaly | | Magovern et al 1996 [[11](#_ENREF_11)] | | Hattler et al 1994 (univariate) [[18](#_ENREF_18)] |
| Cardiopulmonary bypass time | | Higgins et al 1997 [[10](#_ENREF_10)] | |  |
| Catheterisation induced coronary closure | | Magovern et al 1996 [[11](#_ENREF_11)] | |  |
| Diuretic use | | Pitkanen et al 2000 [[1](#_ENREF_1)] | |  |
| Extracardiacarteriopathy | | Huijskes et al 2003 [[3](#_ENREF_3)] | |  |
| Failed percutaneous coronary intervention | | Huijskes et al 2003 [[3](#_ENREF_3)] | |  |
| Hb (see also anaemia) | | Huijskes et al 2003 [[3](#_ENREF_3)] | |  |
| Intra-aortic balloon pump (pre-op) | | Parsonnet et al 1989 [[4](#_ENREF_4)], Hattler et al 1994 [[18](#_ENREF_18)] | |  |
| Inotropes (pre-op) | | Hattler et al 1993 [[18](#_ENREF_18)] | |  |
| Intubation (pre-op) | | Roques et al 1995 [[5](#_ENREF_5)] | |  |
| Left ventricular aneurysm | | Parsonnet et al 1989 [[4](#_ENREF_4)] | |  |
| Liver disease | | Fortescue et al 2001 [[12](#_ENREF_12)] | | Higgins et al 1992 [[6](#_ENREF_6)] |
| Nitrates (IV and pre-op) | | Hattler et al 1994 [[18](#_ENREF_18)] | |  |
| No diseased vessels | | Geraci et al 1993 [[13](#_ENREF_13)], Ivanov et al 2006 [[17](#_ENREF_17)] | | Hattler et al 1994 [[18](#_ENREF_18)] |
| NYHA class | | Pitkanen et al 2000 [[1](#_ENREF_1)], Hattler et al 1994 [[18](#_ENREF_18)] | |  |
| Off-cardiopulmonary bypass procedure | | Janssen et al 2004 [[20](#_ENREF_20)] | |  |
| Pulse | | Geraci et al 1993[[13](#_ENREF_13)], Higgins et al 1997 [[10](#_ENREF_10)] | |  |
| Saphenous vein graft only | | Roques et al 1995 [[5](#_ENREF_5)] | |  |
| Unstable angina or recent/ongoing yocardial infarction | | Tremblay et al 1993 [[14](#_ENREF_14)], Pitkanen et al 2000 [[1](#_ENREF_1)] | | Hattler et al 1994 [[18](#_ENREF_18)], Tuman et al 1992[[7](#_ENREF_7)] |
| Valve: mild pathology | | Janssen et al 2004 [[20](#_ENREF_20)] | |  |
| Ventricular arrhythmias | | Staat et al 1999 [[19](#_ENREF_19)], Roques et al 1995 [[5](#_ENREF_5)] | |  |
| Weight | | Parsonnet et al 1989 [[4](#_ENREF_4)], Higgins et al 1992 [[6](#_ENREF_6)] | |  |
| **Variables not in C-POMS dataset** | | | | |
| ASO lower limbs | | | Pitkanen et al 2000 [[1](#_ENREF_1)] |  |
| Catastrophic states, critical pre-op states | | | Parsonnet et al 1989 [[4](#_ENREF_4)], Huijskes et al 2003 [[3](#_ENREF_3)], Tremblay et al 1993 [[14](#_ENREF_14)] |  |
| Infiltrate on X-ray | | | Geraci et al 1993 [[13](#_ENREF_13)] |  |
| Pulmonary artery pressure | | | Parsonnet et al 1989 [[4](#_ENREF_4)] |  |
| Pulmonary embolectomy | | | Roques et al 1995 [[5](#_ENREF_5)] |  |
| Pulmonary hypertension | | | Tuman et al 1992 [[7](#_ENREF_7)], Huijskes et al 2003 [[3](#_ENREF_3)] | Hattler et al 1994 [[18](#_ENREF_18)] |
| Pulmonary rales | | | Pitkanen et al 2000 [[1](#_ENREF_1)] |  |
| Right heart failure (symptomatic) | | | Staat et al 1999 [[19](#_ENREF_19)] |  |
| Time from failed percutaneous transluminal coronary angioplasty to CABG | | | Hattler et al 1994 [[18](#_ENREF_18)] |  |
| Transplantation | | | Roques et al 1995 [[5](#_ENREF_5)] |  |
| Urea nitrogen | | | Geraci et al 1993 [[13](#_ENREF_13)], Magovern et al 1996 [[11](#_ENREF_11)] |  |
| Valve pressure gradient | | | Parsonnet et al 1989 [[4](#_ENREF_4)] |  |
| Vascular surgery | | | Higgins et al 1992 [[6](#_ENREF_6)] |  |
| Ventricular septal defect (post MI) | | | Roques et al 1995 [[5](#_ENREF_5)] |  |

**REFERENCES**

[1] Pitkanen O, Niskanen M, Rehnberg S, Hippelainen M, Hynynen M. *Intra-institutional prediction of outcome after cardiac surgery: comparison between a locally derived model and the EuroSCORE*. EurJCardiothoracSurg 2000;**18**:703-10.

[2] Wouters SC, Noyez L, Verheugt FW, Brouwer RM. *Preoperative prediction of early mortality and morbidity in coronary bypass surgery*. CardiovascSurg 2002;**10**:500-05.

[3] Huijskes RV, Rosseel PM, Tijssen JG. *Outcome prediction in coronary artery bypass grafting and valve surgery in the Netherlands: development of the Amphiascore and its comparison with the Euroscore*. EurJCardiothoracSurg 2003;**24**:741-49.

[4] Parsonnet V, Dean D, Bernstein AD. *A method of uniform stratification of risk for evaluating the results of surgery in acquired adult heart disease*. Circulation 1989;**79**:I3-12.

[5] Roques F, Gabrielle F, Michel P, de Vincentiis C, David M, Baudet E. *Quality of care in adult heart surgery: proposal for a self-assessment approach based on a French multicenter study*. EurJCardiothoracSurg 1995;**9**:433-39.

[6] Higgins TL, Estafanous FG, Loop FD, Beck GJ, Blum JM, Paranandi L. *Stratification of morbidity and mortality outcome by preoperative risk factors in coronary artery bypass patients. A clinical severity score*. JAMA 1992;**267**:2344-48.

[7] Tuman KJ, McCarthy RJ, March RJ, Najafi H, Ivankovich AD. *Morbidity and duration of ICU stay after cardiac surgery. A model for preoperative risk assessment*. Chest 1992;**102**:36-44.

[8] Tu JV, Jaglal SB, Naylor CD. *Multicenter validation of a risk index for mortality, intensive care unit stay, and overall hospital length of stay after cardiac surgery. Steering Committee of the Provincial Adult Cardiac Care Network of Ontario*. Circulation 1995;**91**:677-84.

[9] Kurki TS, Kataja M. *Preoperative prediction of postoperative morbidity in coronary artery bypass grafting*. AnnThoracSurg 1996;**61**:1740-45.

[10] Higgins TL, Estafanous FG, Loop FD, Beck GJ, Lee JC, Starr NJ *et al.* *ICU admission score for predicting morbidity and mortality risk after coronary artery bypass grafting*. AnnThoracSurg 1997;**64**:1050-58.

[11] Magovern JA, Sakert T, Magovern GJ, Benckart DH, Burkholder JA, Liebler GA *et al.* *A model that predicts morbidity and mortality after coronary artery bypass graft surgery*. JAmCollCardiol 1996;**28**:1147-53.

[12] Fortescue EB, Kahn K, Bates DW. *Development and validation of a clinical prediction rule for major adverse outcomes in coronary bypass grafting*. AmJCardiol 2001;**88**:1251-58.

[13] Geraci JM, Rosen AK, Ash AS, McNiff KJ, Moskowitz MA. *Predicting the occurrence of adverse events after coronary artery bypass surgery*. AnnInternMed 1993;**118**:18-24.

[14] Tremblay NA, Hardy JF, Perrault J, Carrier M. *A simple classification of the risk in cardiac surgery: the first decade*. CanJAnaesth 1993;**40**:103-11.

[15] Eagle KA, Guyton RA, Davidoff R, Ewy GA, Fonger J, Gardner TJ *et al.* *ACC/AHA Guidelines for Coronary Artery Bypass Graft Surgery: A Report of the American College of Cardiology/American Heart Association Task Force on Practice Guidelines (Committee to Revise the 1991 Guidelines for Coronary Artery Bypass Graft Surgery). American College of Cardiology/American Heart Association*. JAmCollCardiol 1999;**34**:1262-347.

[16] Wong DT, Cheng DC, Kustra R, Tibshirani R, Karski J, Carroll-Munro J *et al.* *Risk factors of delayed extubation, prolonged length of stay in the intensive care unit, and mortality in patients undergoing coronary artery bypass graft with fast-track cardiac anesthesia: a new cardiac risk score*. Anesthesiology 1999;**91**:936-44.

[17] Ivanov J, Borger MA, Rao V, David TE. *The Toronto Risk Score for adverse events following cardiac surgery*. CanJCardiol 2006;**22**:221-27.

[18] Hattler BG, Madia C, Johnson C, Armitage JM, Hardesty RL, Kormos RL *et al.* *Risk stratification using the Society of Thoracic Surgeons Program*. AnnThoracSurg 1994;**58**:1348-52.

[19] Staat P, Cucherat M, George M, Lehot JJ, Jegaden O, Andre-Fouet X *et al.* *Severe morbidity after coronary artery surgery: development and validation of a simple predictive clinical score*. EurHeart J 1999;**20**:960-66.

[20] Janssen DP, Noyez L, Wouters C, Brouwer RM. *Preoperative prediction of prolonged stay in the intensive care unit for coronary bypass surgery*. EurJCardiothoracSurg 2004;**25**:203-07.

[21] Fortescue EB, Bates DW, Chertow GM. *Predicting acute renal failure after coronary bypass surgery: cross-validation of two risk-stratification algorithms*. Kidney Int 2000;**57**:2594-602.
