# Supplementary material for: Predictors of total morbidity burden on days 3, 5 and 8 after cardiac surgery
Source: Perioper Med (Lond). 2017 Feb 14;6:2. doi: 10.1186/s13741-017-0060-9 (PMC5307860; doi:10.1186/s13741-017-0060-9)
Supplement: Additional file 2: — Univariate analysis: tier 1 and 2 not predictive of C-POMS summary score on D3, D5 and D8. (DOCX 20 kb) [file 13741_2017_60_MOESM2_ESM.docx]

**Supplementary Table 2: Univariate analysis: Tier 1 and 2 not predictive of C-POMS summary score on D3, D5 and D8.**

|  | **D3 (n=441)** | | **D5 (n=419)** | | **D8 (n=177)** | |
| --- | --- | --- | --- | --- | --- | --- |
| Variable | **Median**  **C-POMS score/Rho** | **p** | **Median**  **C-POMS score/Rho** | **p** | **Median**  **C-POMS score/Rho** | **p** |
| **TIER 1 VARIABLES** |  |  |  |  |  |  |
| **Medical History** |  |  |  |  |  |  |
| Hypertension – Y  - N | 3.0  3.0 | 0.263 | 2.0  2.0 | 0.399 | 3.0  3.0 | 0.435 |
| CVA – Y  - N | 4.0  3.0 | 0.184 | 3.0  2.0 | 0.099 | 4.5  3.0 | 0.702 |
| TIA – Y  - N | 4.0  3.0 | 0.168 | 3.0  2.0 | 0.148 | 4.0  3.0 | 0.212 |
| Peripheral vascular disease – Y  - N | 3.0  3.0 | 0.507 | 2.0  2.0 | 0.500 | 3.5  3.0 | 0.192 |
| Previous cardiac surgery  – Y  - N | 4.0  3.0 | 0.192 | 2.0  2.0 | 0.545 | 3.0  3.0 | 0.885 |
| **Pre-operative measurements** |  |  |  |  |  |  |
| Systolic blood pressure (mmHg) | 0.049 | 0.302 | 0.023 | 0.640 | 0.060 | 0.430 |
| Body mass index quartiles  -1 (0-24.86)  -2 (24.87-27.95)  -3 (27.96-31.33)  -4 (≥31.34) | 3.0  3.0  3.0  3.0 | 0.281 | 2.0  2.0  2.0  2.0 | 0.445 | 3.0  3.0  3.0  3.0 | 0.562 |
| Body mass index (WHO class)  -0-18.49  -18.50-24.99  -25.00-29.99  -≥ 30.0 | 3.0  3.0  3.0  4.0 | 0.088 | 1.0  2.0  2.0  2.0 | 0.228 | 2.0  3.0  3.0  3.0 | 0.795 |
| **TIER 2 VARIABLES** |  |  |  |  |  |  |
| **Medical history** |  |  |  |  |  |  |
| Previous MI -0  -1  -2 | 3.0  3.0  4.0 | 0.240 | 2.0  2.0  3.0 | 0.534 | 3.0  3.0  4.0 | 0.246 |
| History of pulmonary disease  – Y  - N | 4.0  3.0 | 0.071 | 3.0  2.0 | 0.063 | 3.0  3.0 | 0.688 |
| Liver disease – Y (n=1)  - N | 7.0  3.0 | 0.135 | 5.0  2.0 | 0.208 | 4.0  3.0 | 0.553 |
| **Pre-operative measurements** |  |  |  |  |  |  |
| Body surface area | 0.017 | 0.734 | 0.027 | 0.588 | 0.131 | 0.093 |
| **Current medications** |  |  |  |  |  |  |
| Nitrates – Y  - N | 3.0  3.0 | 0.155 | 2.0  2.0 | 0.103 | 3.0  3.0 | 0.854 |
| **Intra-operative** |  |  |  |  |  |  |
| Saphenous vein grafts only – Y  - N | 4.0  3.0 | 0.158 | 2.0  2.0 | 0.147 | 3.0  3.0 | 0.595 |
| **Within 1^st^ 12 hrs after surgery** |  |  |  |  |  |  |
| Inotropes – Y  - N | 3.0  3.0 | 0.407 | 2.0  2.0 | 0.680 | 2.5  3.0 | 0.201 |
| Central venous pressure | 0.069 | 0.149 | 0.064 | 0.188 | 0.120 | 0.110 |
